# Evaluating native-like structures of RNA-protein complexes through the deep learning method

**DOI:** 10.1038/s41467-023-36720-9

**Published:** 2023-02-24

**Authors:** Chengwei Zeng, Yiren Jian, Soroush Vosoughi, Chen Zeng, Yunjie Zhao

**Affiliations:** 1 Institute of Biophysics and Department of Physics, Central China Normal University, Wuhan, 430079 China; 2 Department of Computer Science, Dartmouth College, Hanover, NH 03755 USA; 3 Department of Physics, The George Washington University, Washington, DC 20052 USA

**Keywords:** RNA, Computational biology and bioinformatics, Molecular modelling, Protein structure predictions, Machine learning

## Abstract

RNA-protein complexes underlie numerous cellular processes, including basic translation and gene regulation. The high-resolution structure determination of the RNA-protein complexes is essential for elucidating their functions. Therefore, computational methods capable of identifying the native-like RNA-protein structures are needed. To address this challenge, we thus develop DRPScore, a deep-learning-based approach for identifying native-like RNA-protein structures. DRPScore is tested on representative sets of RNA-protein complexes with various degrees of binding-induced conformation change ranging from fully rigid docking (bound-bound) to fully flexible docking (unbound-unbound). Out of the top 20 predictions, DRPScore selects native-like structures with a success rate of 91.67% on the testing set of bound RNA-protein complexes and 56.14% on the unbound complexes. DRPScore consistently outperforms existing methods with a roughly 10.53–15.79% improvement, even for the most difficult unbound cases. Furthermore, DRPScore significantly improves the accuracy of the native interface interaction predictions. DRPScore should be broadly useful for modeling and designing RNA-protein complexes.

## Abstract (web summary)

RNA-protein docking is a very challenging area. Here, the authors develop a deep-learning based method, DRPScore, to evaluate RNA-protein complexes. DRPScore is robust and consistently performs better than existing methods on representative testing sets.

## Introduction

RNA regulates various biological functions by interacting with proteins, such as DNA repair, RNA splicing, protein synthesis, and gene regulation^1–6^. It is recognized that RNA-protein complexes are involved in many human diseases ranging from neurologic disorders^7,8^ to cancer^9^. Understanding the biological roles of the RNA-protein complex requires a three-dimensional structure^10–12^. Unfortunately, the flexible RNA molecules are challenging to be well-crystallized and determined by X-ray crystallography^13^. Besides, electron microscopy is expensive and time-consuming^14^. The available RNA-protein experimental structures are few due to the technical limitations^15^. Some computational methods can predict the RNA-protein complex by homologous fragment modeling, docking, or molecular dynamics simulation^16–21^. However, it is still challenging to predict the highly accurate RNA-protein complex due to the limited RNA-protein scoring functions^22^.

Several computational methods have been developed to evaluate RNA-protein structures^21,23,24^. These methods can be divided into propensity-based and atomic-level statistical scoring functions^25^. The propensity-based scoring functions statistically analyzed the interface propensity of pairwise nucleotide-residue^26,27^. Then, a potential statistical formula was constructed based on the inverse Boltzmann formula. For example, DARS-RNP^24^ is one coarse-grained propensity-based scoring function introduced by Tuszynska and Bujnicki. DARS-RNP was developed by a reduced representation of protein and RNA. In this reduced representation, amino residues are represented by one to three united atoms^24,28,29^. For RNA representation, two united atoms are used for the backbone and one/two for pyrimidines/purines. DARS-RNP constructed the scoring function through the four terms: the steric clash penalty and dependencies on distances, angles, and sites. Subsequently, Xiao et al.^30^ constructed a novel scoring function, 3dRPC-Score, based on statistical potential energy using the conformation of nucleotide-residue pairs as statistical variables. Unlike DARS-RNP, it used relative RMSD (Root Mean Square Deviation) to assess conformational differences between nucleotide-residue pairs to reflect energy, thus using only one variable. The propensity-based scoring function can consider the pairwise-based nucleotide-residue interactions, but it is challenging to consider conformational changes^21,24^.

The atomic-level statistical scoring functions are the distance-dependent interaction potentials obeying the Boltzmann distribution, which is more discriminative than the propensity-based scoring functions in the native-like structure evaluation. For example, ITScore-PR^23^ is one atomic-level statistical scoring function developed by Huang and Zou. The core idea of ITScore-PR is to improve the interatomic pair potential through iterations by comparing the differences between the predicted and native atomic pairs in the training set. However, a significant challenge for RNA-protein prediction is the conformational change upon binding. ITScore-PR is very effective at bound docking but challenging to deal with unbound docking. Deep learning strategies have been proved helpful for RNA and protein predictions^31–36^. But such techniques are not in widespread use for RNA-protein complex prediction yet. Moreover, the computational structure evaluation methods for unbound complex structures have not yet been developed.

Here, we propose a deep-learning-based RNA-protein complex scoring function to consider structural flexibility explicitly. We use physics-based simulations to generate the training decoys for training the deep-learning-based scoring function. Then, DRPScore is extensively evaluated on the RNA-protein testing sets, including unbound RNA-protein challenges. The results demonstrate significant improvements and are consistently better than the existing methods in selecting native-like RNA-protein complexes. We expect the technique described here to be useful for RNA-protein complex prediction.

## Results

### Testing on the bound RNA-protein testing set

**Fig. 1:**
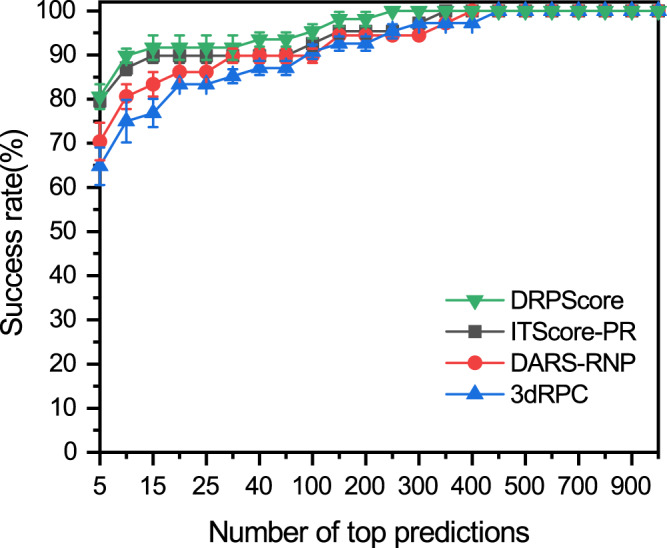
The performance of DRPScore and other scoring functions on the bound-bound testing sets. The average success rates and standard deviations (number = 3 independent tests) of DRPScore (green inverted triangle), ITScore-PR (black square), DARS-RNP (red circle), and 3dRPC (blue triangle) on the bound-bound testing sets. Data are presented as mean values + /- SD. Source data are provided as a Source Data file.

The DRPScore was evaluated on the three generated bound-bound RNA-protein testing sets. Figure 1 shows the average success rates and standard deviations for DRPScore, ITScore-PR^23^, DARS-RNP^24^, and 3dRPC^30^, respectively. Supplementary Figs. 1–3 and Supplementary Data 1–3 show the individual prediction success rates, ranking, and ${I}_{{rmsd}}$. ITScore-PR performs significantly better than DARS-RNP and 3dRPC with success rates from the top 5 to top 30 predictions while achieving similar results with DARS-RNP from top 30 to 1000 predictions. It is noted that DRPScore performs consistently better than ITScore-PR, DARS-RNP, and 3dRPC. The results demonstrate that the DRPScore performs better with an average success rate of 80.56%, compared with 79.63% for ITScore-PR, 70.37% for DARS-RNP, and 64.81% for 3dRPC in the top 5 predictions. When the top 20 predictions were considered, the average success rates of DRPScore increased to 91.67%, compared with 89.81% for ITScore-PR, 86.11% for DARS-RNP, and 83.33% for 3dRPC, respectively. DRPScore can identify the native-like bound-bound RNA-protein structures with high accuracy.

### Testing on the unbound RNA-protein testing set

We further focused on the relatively difficult unbound cases to evaluate whether DRPScore consistently performs better in challenging cases. The unbound RNA-protein testing set was constructed by Huang and Zou^37^. At least one partner of each complex was taken from other complexes or homologous modeling.

**Fig. 2:**
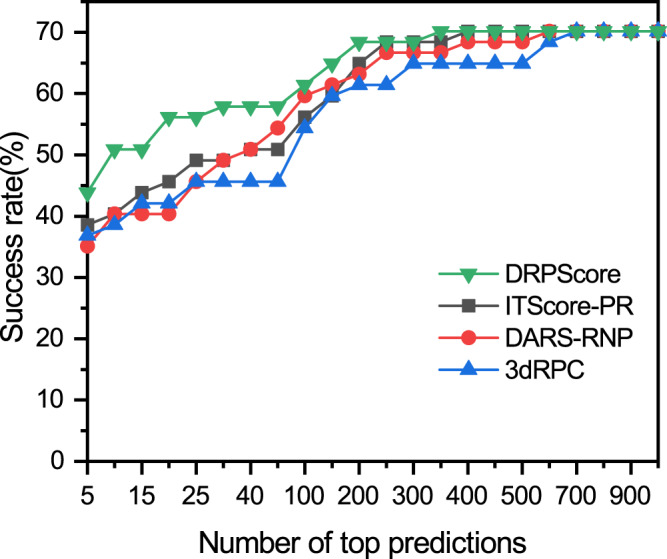
The performance of DRPScore and other scoring functions on the unbound-bound and unbound-unbound testing set. The success rates of DRPScore (green inverted triangle), ITScore-PR (black square), DARS-RNP (red circle), and 3dRPC (blue triangle) on the unbound-bound and unbound-unbound testing sets provided by Huang and Zou^37^. Source data are provided as a Source Data file.

Figure 2 shows the performances of DRPScore, ITScore-PR, DARS-RNP, and 3dRPC for unbound-bound and unbound-unbound tests. The success rate of DRPScore is 43.86% in the top 5 predictions, compared with 38.60% for ITScore-PR, 35.09% for DARS-RNP, and 36.84% for 3dRPC. When the top 20 predictions were considered, the success rate of DRPScore was 56.14%, compared with 45.61% for ITScore-PR, 40.35% for DARS-RNP, and 42.11% for 3dRPC. The ranking and ${I}_{{rmsd}}$ are shown in Supplementary Data 4. We also calculated the score-RMSD scatter plots for the complexes within 8 Å RMSDs (Supplementary Fig. 4). Overall, DRPScore performs significantly better than ITScore-PR, DARS-RNP, and 3dRPC.

**Fig. 3:**
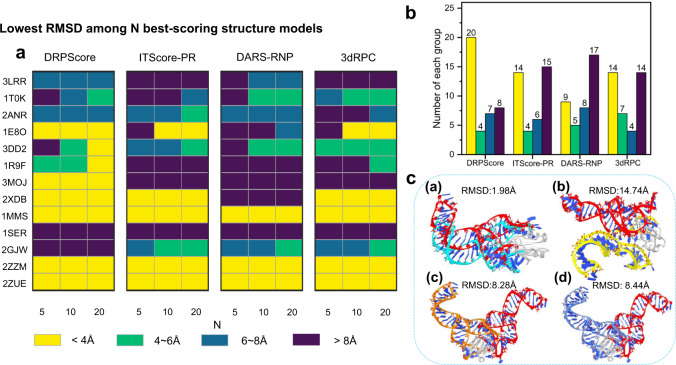
Detailed analysis of native-like structures ranking in the unbound-bound testing set. **a** The N best-scoring structural models for each unbound-bound docking RNA-protein complex for DRPScore, ITScore-PR, DARS-RNP, and 3dRPC in the top 5, 10, and 20 predictions. The RNA-protein complexes are sorted (top to bottom) by the number of nucleotides/residues at the RNA-protein interaction interface. For each scoring function, RNA, and value of N, the lowest RMSD (Root Mean Square Deviation) across structural decoys is recorded. These results are quantized by determining if the RMSD is below 4 Å (yellow), between 4~6 Å (green), between 6~8 Å (blue), or above 8 Å (purple). **b** The statistical prediction results of unbound-bound docking testing sets. **c** One example (PDB ID: 3MOJ) to illustrate the RMSD between the native structure (RNA in red) and the lowest RMSD structures in the top 5 predictions by DRPScore (RNA in cyan), ITScore-PR (RNA in yellow), DARS-RNP (RNA in origin) and 3dRPC (RNA in blue). Source data are provided as a Source Data file.

Figure 3 shows the ranking of unbound-bound RNA-protein models using DRPScore and the other three leading scoring functions. Three complexes were not considered because all the docking decoys’ RMSDs were >4 Å. We selected the best-scoring models for each scoring function in the top 5, 10, and 20 models. For each RNA-protein complex, we recorded the lowest RMSD across the models. We quantized the predictions into 4 categories by RMSD ranking: below 4 Å (colored in yellow), between 4 Å and 6 Å (colored in green), between 6 Å and 8 Å (colored in blue), and above 8 Å (colored in purple). Figure 3a shows RNA-protein complexes ranking by the number of nucleotides/residues at the interaction interface. An interesting observation is that DRPScore performs best on RNA-protein models when the number of interface interaction nucleotides/residues is relatively small. The other three scoring functions give unsatisfactory results. Figure 3b shows that DRPScore has the largest proportions of yellow and green areas compared to the other three scoring functions. The statistics of prediction numbers with RMSD <4 Å are 20 for DRPScore, compared with 14 for ITScore-PR, 9 for DARS-RNP, and 14 for 3dRPC, respectively. Moreover, predictions of DRPScore with RMSD >8 Å are only 8 cases, compared with 15 for ITScore-PR, 17 for DARS-RNP, and 14 for 3dRPC, respectively. For example, the lowest RMSDs in the top 5 predictions of the Bacillus subtilis YxiN protein complexed with a fragment of 23 S ribosomal RNA (PDB ID: 3MOJ) are *I*_*rmsd*_ = 1.98 Å for DRPScore, compared with *I*_*rmsd*_ = 14.74 Å for ITScore-PR, *I*_*rmsd*_ = 8.28 Å for DARS-RNP, *I*_*rmsd*_ = 8.44 Å for 3dRPC, respectively (Fig. 3c). Together, DRPScore can accurately identify the RNA-protein interface features when the number of interface interactions is relatively small. However, the other scoring functions are too sensitive to interaction changes leading to unsatisfactory results.

**Fig. 4:**
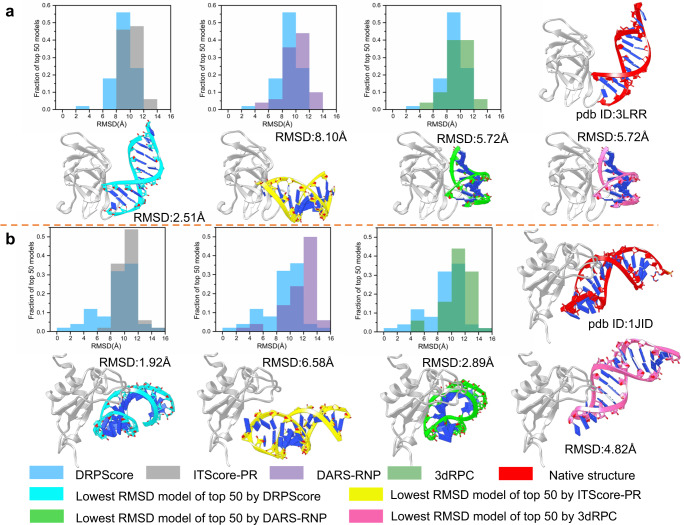
Two examples of native-like structures ranking analysis. **a** Unbound-bound (PDB ID: 3LRR) and (**b**) unbound-unbound (PDB ID: 1JID) testing sets. The histograms (left to right) are the RMSD (Root Mean Square Deviation) distributions relative to native structure in the top 50 predictions of DRPScore (blue) compared with ITScore-PR (gray), DARS-RNP (purple), 3dRPC (green), respectively. DRPScore shows a shift in the distribution of the predictions toward lower RMSDs. The lowest RMSD models in the top 50 predictions by DRPScore (RNA in cyan) are more similar to native complexes (RNA in red) than ITScore-PR (RNA in yellow), DARS-RNP (RNA in green) and 3dRPC (RNA in pink), respectively. Source data are provided as a Source Data file.

To further evaluate the performance of poor sampling structures, we analyzed the top 50 best-scoring structure models of human RIG-I CTD bound to a dsRNA (PDB ID: 3LRR) as an example (Fig. 4a). The lowest RMSD model of DRPScore is 2.51 Å, compared with 8.10 Å for ITScore-PR, 5.72 Å for DARS-RNP, and 5.72 Å for 3dRPC, respectively. DRPScore achieved an average RMSD of 8.94 Å (colored in blue), better than RMSDs of around 10.0 Å in the other three scoring functions (colored in gray, purple, and green). Together, DRPScore resulted in a shift in the distribution of the predictions toward lower RMSDs.

### Physics-based interaction contributions

The existing RNA-protein complex evaluation methods are all statistical potential functions based on Boltzmann’s formula^23,24,30^. DRPScore used an alternative approach to evaluate the RNA-protein complex by deep learning. Thus, DRPScore can accurately extract more features from the individual frames in the training sets. The separate local features can infer the interface interactions. For example, we calculated the interface hydrogen bonds of the Bacillus subtilis YxiN protein complexed with a 23 S ribosomal RNA fragment (PDB ID: 3MOJ) using HBPULS^38^. The results show that the lowest RMSD structure in the top 5 predictions evaluated by DRPScore contains 8 hydrogen bonds at the RNA-protein interaction interface, compared with 17 for ITScore-PR, 10 for DARS-RNP, and 16 for 3dRPC, respectively (Supplementary Table 1). However, it is noted that 62.5% of the hydrogen bonds identified by DRPScore agree with the experimental structure. None of the native hydrogen bonds are identified by ITScore-PR, DARS-RNP, and 3dRPC.

Learning the global features is much more challenging due to the lack of individual frames. Our approach contains an extra dimension to consider the relations between the secondary structure frames. Therefore, DRPScore can consider the global features, including α-helix and β-sheet for protein, hairpin loop, internal loop, bulge loop, junction, and pseudoknot for RNA. For example, we calculated the secondary structures of protein and RNA using PSIPRED^39,40^ and forna^41^ (Supplementary Figs. 5 and 6). The results show that 25% of hydrogen bonds identified by DRPScore are loop-helix secondary structure interactions, compared with 0.0% for ITScore-PR, 0.0% for DARS-RNP, and 12.5% for 3dRPC (Supplementary Table 1). The secondary structure interactions on the RNA-protein interface would improve the assessment accuracy.

### Advances compared to the traditional deep learning model

DRPScore applied the 4DCNN to reduce the mean squared errors between the true and predicted results. In the back-propagation-based mini-batch gradient descent optimization algorithm, the learning rate of 4DCNN was initially set to 0.0001. The training processes stopped when the loss decreased to approach 0 and stabilized. The training steps were 20000, around 6 seconds per iteration, and 6.6 GB for ‘---bacth_size 1’ on the GeForce RTX 3070. Finally, a 12500-step model was selected. DRPScore takes about 8 min to evaluate 1000 RNA-protein complex structures.

We compared our deep-learning-based model with the traditional 3DCNN model. 3DCNN utilizes three dimensions to extract and transfer the X, Y, and Z coordinates information to one image. There are no connections between any two images. Thus, 3DCNN provides and learns the local but without global structural features. In other words, it captures the intra-nucleotide/residue information while ignoring the inter-nucleotide/residue interactions. However, the representations learned by our model capture both the intra- (local) and inter-nucleotide/residue (global) information. This is done by adding convolutional layers at the sequence dimension, each layer gradually modeling a longer range of interactions between the nucleotides/residues. Therefore, our model can provide and learn both local and global structural features, including secondary structure interactions. Supplementary Fig. 7 shows the average success rates and standard deviations for DRPScore, 3DCNN, ITScore-PR, DARS-RNP, and 3dRPC on the three generated bound-bound RNA-protein testing sets, respectively. However, 3DCNN is not able to identify the native-like RNA-protein complex accurately.

## Discussion

RNA-protein structure evaluation is still a relatively unexplored research field. The previous efforts focused on the RNA-protein rigid-body docking without consideration of the structural flexibility. A critical bottleneck is sampling the dynamical conformations that RNAs or proteins form when interacting. In the case of fully flexible unbound-unbound docking, the interaction interface has changed dramatically. And the scoring functions never learned similar structures before. Supplementary Fig. 8 shows the success rate of DRPScore and the three other scoring functions in the unbound-unbound cases in the testing set II when considering the top10, top20, top30, and top40, respectively. Although the performance of the four scoring functions is not satisfactory compared with bound-bound and unbound-bound cases, DRPScore still achieves better results. For example, when the top 20 predictions are considered, the average success rate of DRPScore is 58.5%, compared with 51.2% for ITScore-PR, 48.8% for DARS-RNP, and 46.3% for 3dRPC, respectively. Figure 4b shows the Human SRP19 complex with SRP RNA (PDB ID: 1JID) as one unbound-unbound RNA-protein example. DRPScore achieves an average RMSD of 8.85 Å (colored in blue), better than RMSDs of around 11.0 Å in the other three scoring functions (colored in gray, purple, and green). The lowest RMSD model in the top 50 predictions of DRPScore is 1.92 Å, compared with 6.58 Å for ITScore-PR, 2.89 Å for DARS-RNP, and 4.82 Å for 3dRPC, respectively.

To test the robustness of DRPScore, we also provided the performance of DRPScore compared with ITScore-PR, DARS-RNP, and 3dRPC on the entire and 0.8 sequences redundancy cutoff for testing set II (Supplementary Figs. 9 and 10)^42–44^. DRPScore still shows lower RMSDs and performs better than the other three scoring functions (ITScore-PR, DRPScore, and 3dRPC) for all testing sets at different redundancy levels. Traditional scoring functions based on statistical potential energy use native structures to learn features of RNA-protein interactions. DRPScore samples many native-like decoys to consider the RNA-protein dynamical features implicitly. As a result, the performance of the DRPScore in the unbound test is better than the other three scoring functions. We will further consider the structural flexibility, such as adding molecular dynamics simulation to consider the flexibility of RNA-protein complexes explicitly.

In summary, we have developed an efficient scoring function for evaluating RNA–protein complexes using a deep-learning-based method. DRPScore has been extensively assessed on its ability to identify native-like structures of RNA-protein complexes on different diverse testing sets. Compared with other available methods, DRPScore showed success rates as high as 80.56% (91.67%) for bound docking and 43.86% (56.14%) for unbound docking if the top 5 (20) predictions were considered. The significant improvements indicate that DRPScore resolves critical flexibility in the structural evaluation of an RNA-protein complex. We expect the method to be helpful for the RNA-related prediction and drug development.

## Methods

### Convolution neural network for RNA-protein complex scoring function

**Fig. 5:**
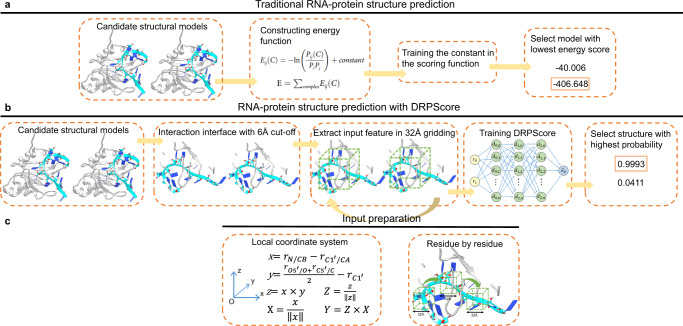
Flow charts comparing DRPScore and traditional scoring functions. **a** The steps of traditional scoring function based on statistical potential energy to evaluate decoys. **b** The steps of DRPScore to evaluate the decoys. **c** The local coordinate framework construction in the generated 32 Å grid.

Previous RNA-protein complex scoring functions used statistical scoring functions to identify the native-like structures (Fig. 5a). The statistical potential function assumes that the distributions of different native structural features obey the Boltzmann distribution. Then, these methods calculated the probability of the interface interactions to construct energy function and identified a native-like complex structure with the lowest energy. Instead of using the entire RNA-protein structure, DRPScore focuses on the RNA-protein interaction interface within a 6 Å distance (Fig. 5b). First, we extracted the RNA-protein interface structures with a 6 Å cut-off. Second, we utilized 85 atom types with mass and charge in the RNA nucleotides (Supplementary Data 5 and Supplementary Table 2) and 225 atom types with mass and charge in the protein residues (Supplementary Data 6 and Supplementary Table 2) to consider the atomic-level interactions. Then, we fed the interaction interface information with the accumulations of the occupation number, mass, and charge of the atoms in the grid to a convolution neural network. A 32 Å grid was created on each nucleotide and residue with a local cartesian coordinate specified by atoms^36^. The **X**, **Y**, and **Z** axes were determined by Eqs. 1–6 where $\,{{{{{{\bf{r}}}}}}}_{{{{{{\bf{N}}}}}}{{{{{\boldsymbol{/}}}}}}{{{{{\bf{CB}}}}}}},\,{{{{{{\bf{r}}}}}}}_{{{{{{\bf{C}}}}}}{{{{{{\bf{1}}}}}}}^{{\prime} }{{{{{\boldsymbol{/}}}}}}{{{{{\bf{CA}}}}}}},\,{{{{{{\bf{r}}}}}}}_{{{{{{\bf{O}}}}}}{{{{{{\bf{5}}}}}}}^{{\prime} }{{{{{\boldsymbol{/}}}}}}{{{{{\bf{O}}}}}}}$ and ${{{{{{\bf{r}}}}}}}_{{{{{{\bf{C}}}}}}{{{{{{\bf{5}}}}}}}^{{\prime} }{{{{{\boldsymbol{/}}}}}}{{{{{\bf{C}}}}}}}$ stand for the vectors pointing from the origin in the global coordinate system to the atom N, CB, C1', CA', O5', O', C5' and C, respectively. As expected, the convolution neural network approach learned much more features than the statistical scoring function.

1 $${{{{{\bf{x}}}}}}={{{{{{\bf{r}}}}}}}_{{{{{{\bf{N}}}}}}/{{{{{\bf{CB}}}}}}}-{{{{{{{\bf{r}}}}}}}_{{{{{{{\bf{C}}}}}}{{{{{\bf{1}}}}}}}^{{\prime} }}}_{/{{{{{\bf{CA}}}}}}}$$

2 $${{{{{\bf{y}}}}}}=\frac{{{{{{{\bf{r}}}}}}}_{{{{{{\bf{O}}}}}}{{{{{{\bf{5}}}}}}}^{{\prime} }/{{{{{\bf{O}}}}}}}+{{{{{{\bf{r}}}}}}}_{{{{{{\bf{C}}}}}}{{{{{{\bf{5}}}}}}}^{{\prime} }/{{{{{\bf{C}}}}}}}}{2}-{{{{{{\bf{r}}}}}}}_{{{{{{\bf{C}}}}}}{{{{{{\bf{1}}}}}}}^{{\prime} }/{{{{{\bf{CA}}}}}}}$$

3 $${{{{{\bf{z}}}}}}={{{{{\bf{x}}}}}}\times {{{{{\bf{y}}}}}}$$

4 $${{{{{\bf{X}}}}}}=\frac{{{{{{\bf{x}}}}}}}{{||}{{{{{\bf{x}}}}}}{||}}$$

5 $${{{{{\bf{Z}}}}}}=\frac{{{{{{\bf{z}}}}}}}{{||}{{{{{\bf{z}}}}}}{||}}$$

6 $${{{{{\bf{Y}}}}}}={{{{{\bf{Z}}}}}}\times {{{{{\bf{X}}}}}}$$

DRPScore used a 4D convolution neural network approach to train and identify the native-like structures. The input of DRPScore is the RNA-protein complex nucleotides/residues at the interaction interface with a 6 Å distance. The output of the DRPScore is the potential scores to evaluate the native-like RNA-protein structures. We compared the 4D convolution neural network with a 3D convolution neural network. In the pre-processing procedures, each RNA is represented by a tensor of shape $1\times 3\times L\times (H\times W\times D)$, where 3 is the number of features: the accumulations of the occupation number, mass, and charge of the atoms in the grid box, *L* = 128 is the maximum length of RNA sequences, *H*, *W*, *D* denote height, width and depth of a 3D cube for each nucleotide in the RNA sequence. In this study, we set *H* = *W* = *D* = 32^36^.

3D convolution neural network approach. The 3D approach processes each nucleotide independently to generate local representations and then applies an average pooling at the end to generate a global representation for the sequence. Concretely, for a sequence comprised of 128 nucleotides, the shape of the input is $3\times 128\times (32\times 32\times 32)$ (3 is the number of channels or features *C*, 32x32x32 is the spatial dimension $H\times W\times D$, and 128 is the number of nucleotides in the sequence); each nucleotide ${{seq}}_{i}$ is processed independently by the 3D convolutional module:

7 $${O}_{L}={Conv}3D({O}_{L-1})$$

Where,${O}_{L-1}$ is a tensor of shape ${C}_{L-1}\times {H}_{L-1}\times {W}_{{{{{{\rm{L}}}}}}-1}\times {D}_{L-1}$ being the representation of an RNA nucleotide at $L-1$ layer of the neural network (e.g., $3\times 32\times 32\times 32$ at layer 0, which is the initial representation of the RNA nucleotide). Conv3D projects channel ${C}_{L-1}$ to ${C}_{L}$ and down-samples the spatial dimension. The output representation of the RNA nucleotide at layer $L$ (i.e., ${O}_{L}$) is a tensor of shape ${C}_{L}\times {H}_{L-1}/2\times {W}_{L-1}/2\times {D}_{L-1}/2$ (e.g.,$64\times 16\times 16\times 16$ at layer 1). The down-sampling enables more compact representations of RNAs and increasing the number of channels at each layer of CNN allows for a larger number of and more expressive features to be learned. After applying *N* layers of Conv3D modules, at the last layer, each nucleotide of the RNA, ${{seq}}_{i}$, is represented by a tensor ${O}_{{L}_{N}}^{{{seq}}_{i}}$ with shape $1024\times 1\times 1\times 1$, where 1024 is the number of channels (i.e., features) in the last layer. The spatial dimension of the representation is down-sampled to $1\times 1\times 1$. Finally, a global representation of the RNA sequence can be generated by averaging the individual representations of each nucleotide: ${O}_{{overall}}=(\frac{1}{{Len}})\times \,{\sum }_{i\in {Len}}{O}_{{L}_{N}}^{{{seq}}_{i}}$. This representation is general-purpose and can be used for both classification and regression tasks for the RNA sequence by adding a single linear layer at the end of the model.

**Fig. 6:**
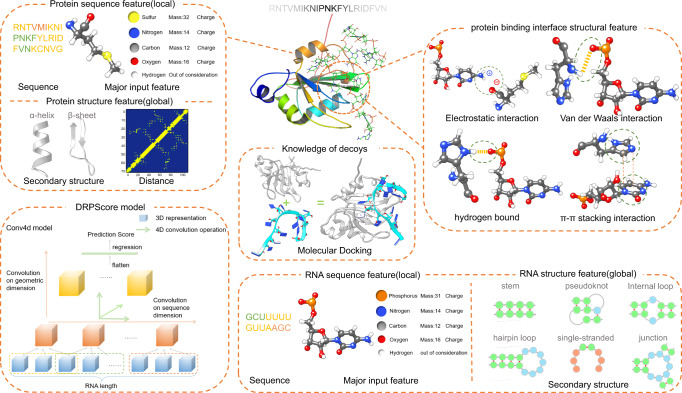
The input features of the DRPScore model. DRPScore considers both local and global features from the RNA-protein complex. The local features are sequence, mass, and charge for each atom. The global features are secondary structures, nucleotide-residue distances, and interface interactions.

4D convolution neural network approach. Though 3D CNN for modeling RNA has shown success in some tasks^36^, it importantly ignores the sequential nature of RNAs. By naively averaging the independent representations of individual nucleotides to generate a global representation, crucial information about the interactions of nucleotides may be lost. Our proposed 4D approach addresses this shortcoming by incorporating an additional convolution operation at the sequential dimension. In other words, our method captures not only the spatial information, but also the sequential information (i.e., interactions between the nucleotides/residues). Our Conv4D uses a non-overlapping moving window of size 3 nucleotides/residues (i.e., a kernel size of 3) to capture interactions between the nucleotides/residues at each layer of the convolution. By using multi-layer CNNs, we can capture the interactions between more distant nucleotides/residues since the input to each layer is the output of the convolution of the last layer. As can be seen in the bottom left panel of Fig. 6, the deeper the CNN, the more long-range interactions can be learned. For instance, in a two-layer CNN, the first layer would capture the interactions between 3 consecutive nucleotides/residues while the second layer would capture the higher-level interactions between each of the 3-nucleotide/residue segments. Thus, at the second layer, we are effectively modeling the nucleotides/residues in RNA-protein complex with distance of 6. Further stacking of multi-layer CNN allows modeling of nucleotides/residues with longer distances (in Computer Vision, this is called increasing the receptive field).

Specifically, our network has six layers, with the last being a fully connected layer for classification. Each of the first six layers has a Conv4d module, a BatchNorm module (optional), and a MaxPooling module. The number of channels in these Conv4d modules are [64, 128, 256, 512, 512]. The strides in these Conv4d modules are [2,2,2,1,1], i.e., the effective length of the RNA-protein complex features is reduced by half in each of the first three blocks (and kept the same in the last 2 blocks). All the pooling modules use a kernel size of two and a stride of two, i.e., each pooling module reduces the effective spatial dimensions (height, width, depth) by half. The last pooling is a global average pooling that reduces the spatial dimensions to one. Thus, the final representation of an RNA-protein complex is a vector of size 8,192. For example, for an RNA-protein complex initially represented by a tensor ${O}_{0}$ of shape $1\times 3\times 128\times 32\times 32\times 32$, we apply a 4D convolution with stride 2 at the sequential dimension:

8 $${O}_{1}={Conv}4D({O}_{0})$$

Where ${O}_{1}$ has shape $1\times 64\times 64\times 32\times 32\times 32$(the number of channels increases from 3 to 64, the effective length of RNA-protein complex reduces from 128 to 64). Note that the calculation of ${O}_{1}[:,:,0,:,:,:]$ relies on ${O}_{0}[:,:,0:2,:,:,:]$. We further apply an additional Max-pooling, i.e., ${O}_{1}={MaxPool}({O}_{1})$., reducing the spatial dimension from 32x32x32 to 16x16x16. Moving forward to next layer:

9 $${O}_{2}={Conv}4D({O}_{1})$$

Where ${O}_{2}$ has shape $1\times 128\times 32\times 16\times 16\times 16$(the number of channels increases from 64 to 128, the effective length of RNA-protein complex decreases from 64 to 32). Note that the calculation of ${O}_{2}[:,:,0,:,:,:]$ relies on ${O}_{1}[:,:,0:2,:,:,:]$, which effectively relies on ${O}_{0}[:,:,0:6,:,:,:]$. Thus, at the second layer, we are effectively modeling the residues in RNA-complex with distance of 6. Again, after applying Max-pooling i.e., ${O}_{2}={MaxPool}({O}_{2})$, the output feature has a shape $1\times 128\times 32\times 8\times 8\times 8$. Similarly, the output ${O}_{3}$ and ${O}_{4}$ have shape of $1\times 256\times 16\times 4\times 4\times 4$ and $1\times 512\times 16\times 2\times 2\times 2$, respectively.

Finally, at the last layer, we have a tensor ${O}_{{L}_{N}}$ (${O}_{5}$) with shape $512\times 16\times 2\times 2\times 2$, which we further apply an adaptive pooling at the spatial dimension to get the final overall representation of RNA-protein complex:

10 $${O}_{{overall}}=\frac{1}{H}\frac{1}{W}\frac{1}{D}\mathop{\sum}\limits_{i\in H}\mathop{\sum}\limits_{j\in W}\mathop{\sum}\limits_{k\in D}{O}_{{L}_{N}}[i,j,k]$$

Where ${O}_{{overall}}$ has the shape $8192\times 1\times 1\times 1$ (after flattening $512\times 16$). Similar to the 3D convolution neural network, this representation is general-purpose and can be used for both classification or regression tasks for the RNA-protein complex sequence by adding a single regression or classification linear layer at the end of the model.

The 4DCNN in this work captures the sequence, secondary structural characteristics, and tertiary structural characteristics of protein and RNA (Fig. 6). The sequence, mass, and charge of heavy atoms are considered as local features in 4DCNN. On the other hand, the secondary structure (α-helix and β-sheet for protein, stem, pseudoknot, internal loop, hairpin loop, single-stranded, and junction for RNA) and distances between each nucleotide and residue are considered as global features in 4DCNN. In addition, the interactions of the RNA-protein binding interface are also fully extracted, including electrostatic interaction, Van der Waals interaction, hydrogen bound, and π-π stacking interaction.

### Training sets

To construct a diverse training dataset of RNA-protein complex structures, we extracted 951 available RNA-protein complex structures from the NDB database (before July 13, 2022) with the search options “only RNA and Protein” and “Resolution cutoff 3.5 Å (X-ray)”^45,46^. Second, we removed the short RNAs with lengths of <10 nucleotides. Third, we considered the cases with no more than six chains of protein or RNA as described in ITScore-PR^23^. Fourth, we removed the RNA redundancy by 0.95 sequence similarity cutoff as RASP^47^ and DRNA^11^ using CD-HIT^42–44^. Finally, we obtained a non-redundant RNA-protein dataset with 346 structures. We randomly selected 277 RNA-protein complex structures for training from the 346 non-redundant RNA-protein structures (Supplementary Data 7). The remaining RNA-protein complexes are further processed to build bound-bound testing sets.

We used 3dRPC^21,30,48^ to generate RNA-protein structural decoys. 3dRPC first generates the RNA-protein complex by the RPDOCK algorithm and then evaluates the structures by RPRANK. For each RNA-protein complex in the training set, 10,000 decoys were generated using the command of ‘3dRPC -mode 9 -system 8 -par RPDOCK.par’. Then, we calculated the RMSDs of the complex structures using the following command ‘3dRPC –mode 2 –system 0 –par RMSD.par’. Finally, we selected the top 500 structures from 10,000 decoys by RMSD ranking. Thus, there are 1 native structure and 500 docking structures for each RNA-protein complex.

### Testing sets

We tested DRPScore with two independent RNA-protein docking testing sets. The unbound structure is defined as a structure in free form or being a binding partner in a different complex^37^. Thus, the definition of a “bound-bound” structure is that two binding partners are taken from the same complex structure. The “unbound-bound” structure is that one of the two binding partners (RNA or protein) is either in apo form or taken from another complex. The “unbound-unbound” refers to structures that both binding partners (RNA and protein) that are either in apo form or taken from a different complex.

Testing set I is the non-redundant bound-bound RNA-protein docking benchmark. We randomly selected 36 RNA-protein complexes from the remaining non-redundant RNA-protein complexes (Supplementary Data 8). We generated three bound-bound RNA-protein sets for a fair comparison. Then, we generated 1000 decoys for each RNA-protein complex in those three sets by 3dRPC^21,30,48^.

Testing set II is the non-redundant unbound RNA-protein docking benchmark provided by Huang and Zou^37^. We removed the redundancy between training and testing sets II by 0.95 sequence similarity cutoff using CD-HIT. Thus, this benchmark remains 57 RNA-protein unbound complex structures, which consist of 41 unbound-unbound complexes and 16 unbound-bound complexes (Supplementary Data 9). For each RNA-protein complex in this benchmark, 1000 decoys were generated by 3dRPC^21,30,48^ using the command of ‘3dRPC -mode 9 -system 8 -par RPDOCK.par’. The relative RMSDs between decoys and native complex structures were calculated using the command of ‘3dRPC –mode 2 –system 0 –par RMSD.par’. The maximum, minimum, and average RMSD were provided in Supplementary Data 10–13.

For RNA-protein complexes ranking, ITScore-PR uses the command of ‘itscorepr protein.pdb RNA.pdb -nomin’, DARS-RNP uses the command of ‘python DARS_potential_v3.py -s complex.pdb’, and 3dRPC uses the command of ‘3dRPC -mode 8 -system 9 -par scoring.par’, respectively.

### Criteria for the assessment of the prediction quality

The quality of the RNA-protein complex prediction is evaluated by the CAPRI criterion^49,50^. The ${I}_{{rmsd}}$ is the interface RMSD between the native and predicted structures after the superposition of corresponding proteins. The definition of the RMSD is

11 $${RMSD}=\sqrt{\frac{1}{N}{\sum}_{i}({\left|{\vec{X}}_{{Ai}}-{\vec{X}}_{{Bi}}\right|}^{2}+{\left|{\vec{Y}}_{{Ai}}-{\vec{Y}}_{{Bi}}\right|}^{2}+{\left|{\vec{Z}}_{{Ai}}-{\vec{Z}}_{{Bi}}\right|}^{2})}$$

where X, Y, and Z are the native and predicted structure coordinates. N is the total number of atoms. All the RNA-protein superimposition and RMSD calculations were performed by 3dRPC^21,30,48^. We define the RNA-protein complex as successfully predicted if the ${I}_{{rmsd}}$ between the predicted and native complexes is less than or equal to 4.0 Å.

We also analyzed the RNA-protein interface’s hydrogen bond and secondary structure interactions. We used HBPLUS^38^ to identify intermolecular hydrogen bonds between the RNA and protein molecules. A maximum donor-acceptor distance of 3.35 Å and a maximum hydrogen-acceptor distance of 2.7 Å were used to define a hydrogen bond. Protein often folds into the secondary structure of α-helix and β-sheet while RNA contains the single-strand, stem, pseudoknot, internal loop, hairpin loop, and junctions. The PSIPRED^39,40^ and forna^41^ were used to identify the protein and RNA secondary structures.

### Reporting summary

Further information on research design is available in the Nature Portfolio Reporting Summary linked to this article.

## Supplementary information

Supplementary Information

Description of Additional Supplementary Files

Supplementary Data 1-13

Reporting Summary

## Data Availability

A full list with links of the PDB codes used in this study is available in supplementary data 7–9. All PDB data sets used in this paper can be downloaded from Nucleic Acid Database (http://ndbserver.rutgers.edu/) and Zoulab (http://zoulab.dalton.missouri.edu/RNAbenchmark/). The data that supports the findings of this study, including scoring function, training set, testing sets, and examples, are available to download at https://github.com/Zhaolab-GitHub/DRPScore_v1.0. Source data are provided with this paper.

## Source data

Source Data

## Code availability

The DRPScore code is freely available for academic or non-commercial users via https://github.com/Zhaolab-GitHub/DRPScore_v1.0^51^.
