# Supplementary material for: Evaluating native-like structures of RNA-protein complexes through the deep learning method
Source: Nat Commun. 2023 Feb 24;14:1060. doi: 10.1038/s41467-023-36720-9 (PMC9958188; doi:10.1038/s41467-023-36720-9)

Supplementary Information:

Evaluating native-like structures of RNA-protein complexes through the deep learning method

Chengwei Zeng[1+], Yiren Jian[2+], Soroush Vosoughi[2], Chen Zeng[3], and Yunjie Zhao[1]*

[1]Institute of Biophysics and Department of Physics, Central China Normal University, Wuhan, 430079, China

[2]Department of Computer Science, Dartmouth College, Hanover, NH 03755, USA

[3]Department of Physics, The George Washington University, Washington, DC 20052, USA

[+]These authors contributed equally.

*Correspondence:

Yunjie Zhao. Email: yjzhaowh@mail.ccnu.edu.cn

Supplementary Table 1. The interface hydrogen bond interactions of the lowest RMSD model (PDB ID:3MOJ) in the top 5 predictions ranking by the four scoring functions. The 'Native' refers to the hydrogen bond that occurs in the native structure, and the 'loop-helix' refers to whether the nucleotide-residue pair that has a hydrogen bond interaction is in the loop region of an RNA and the helix region of a protein.

Scoring function	Number/Type	Distance	Native	loop-helix
DRPScore	413LYS-549G	2.68	×	×
	555U-420ASP	2.42	√	√
	555U-420ASP	2.49	√	√
	553G-436ILE	2.59	√	×
	553G-437GLY	2.09	√	×
	439ILE-553G	2.62	√	×
	473LYS-570G	3.02	×	×
	473LYS-571U	2.86	×	×
ITScore-PR	510C-409ASN	2.58	×	×
	510C-409ASN	3.47	×	×
	409ASN-511U	2.73	×	×
	413LYS-579C	2.57	×	×
	579C-443ASP	3.30	×	×
	579C-443ASP	2.27	×	×
	510C-444ASN	2.64	×	×
	444ASN-579C	1.89	×	×
	463LYS-549G	3.08	×	×
	463LYS-549G	2.77	×	×
	561U-464ASN	3.50	×	×
	560A-464ASN	2.75	×	×
	466THR-562U	2.59	×	×
	466THR-562U	2.23	×	×
	562U-466THR	2.23	×	×
	471GLN-548U	2.58	×	×
	548U-472LEU	2.95	×	×
DARS-RNP	409ASN-557G	3.25	×	×
	552U-413LYS	3.31	×	×
	550G-444ASN	1.84	×	×
	444ASN-551C	2.18	×	×
	447TYR-508G	2.21	×	×
	447TYR-509G	2.81	×	×

	460LYS-571U	2.91	×	×
	466THR-555U	1.74	×	×
	555U-467VAL	3.10	×	×
	473LYS-550G	3.26	×	×
3dRPC	551C-409ASN	2.83	×	×
	409ASN-550G	2.73	×	×
	409ASN-557G	3.18	×	×
	418ALA-553G	1.63	×	√
	419VAL-553G	2.86	×	√
	440THR-508G	2.49	×	×
	440ASN-550G	3.09	×	×
	550G-444ASN	1.25	×	×
	447TYR-508G	3.16	×	×
	508G-447TYR	3.31	×	×
	447TYR-509G	1.83	×	×
	466THR-555U	1.41	×	×
	467VAL-555U	2.59	×	×
	555U-467VAL	2.50	×	×
	473LYS-549G	2.78	×	×
	473LYS-550G	3.37	×	×

Supplementary Table 2. The atom types and mass on the RNA nucleotides and protein residues.

Atom type	Mass
C	12.0107
O	15.9994
N	14.0067
P	30.9738
S	32.0655

Supplementary Figure 1. The performance of DRPScore and other scoring functions on the first independent bound-bound testing sets. The success rates of the first independent bound-bound testing set for DRPScore (green inverted triangle), ITScore-PR (black square), DARS-RNP (red circle), and 3dRPC (blue triangle). Source data are provided as a Source Data file.

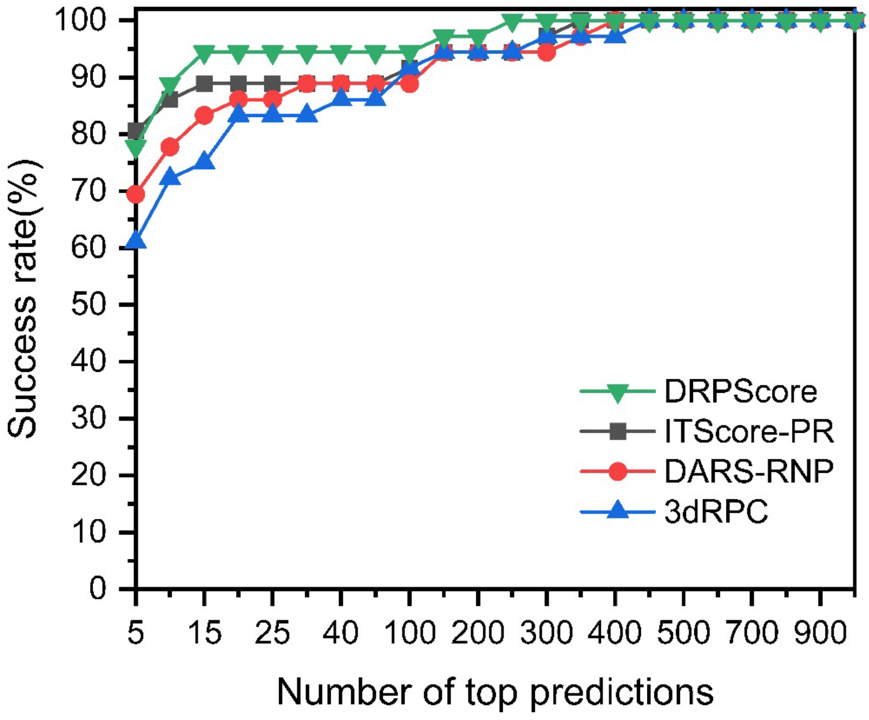

Supplementary Figure 2. The performance of DRPScore and other scoring functions on the second independent bound-bound testing sets. The success rates of the second independent bound-bound testing set for DRPScore (green inverted triangle), ITScore-PR (black square), DARS-RNP (red circle), and 3dRPC (blue triangle). Source data are provided as a Source Data file.

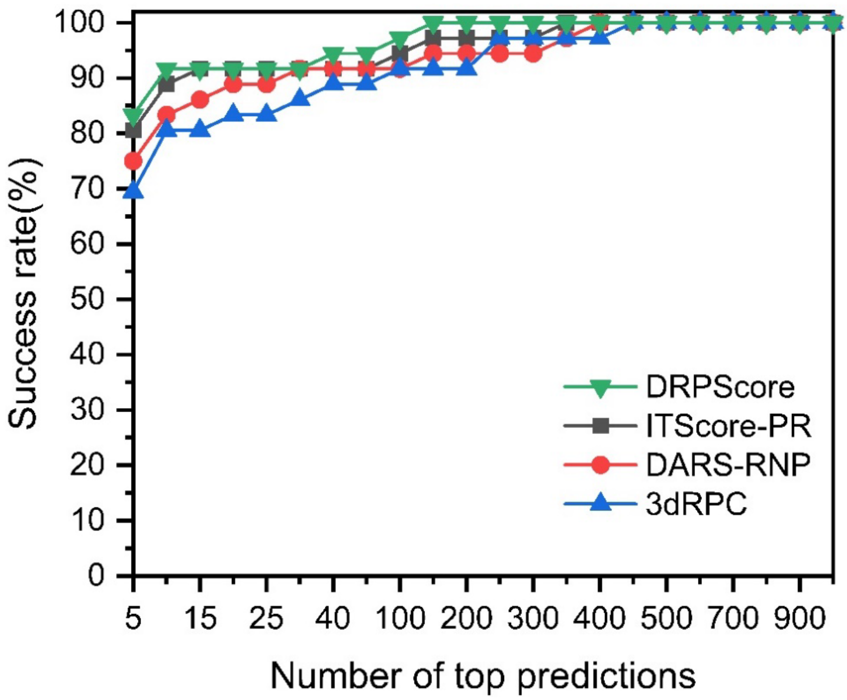

Supplementary Figure 3. The performance of DRPScore and other scoring functions on the third independent bound-bound testing sets. The success rates of the third independent bound-bound testing set for DRPScore (green inverted triangle), ITScore-PR (black square), DARS-RNP (red circle), and 3dRPC (blue triangle). Source data are provided as a Source Data file.

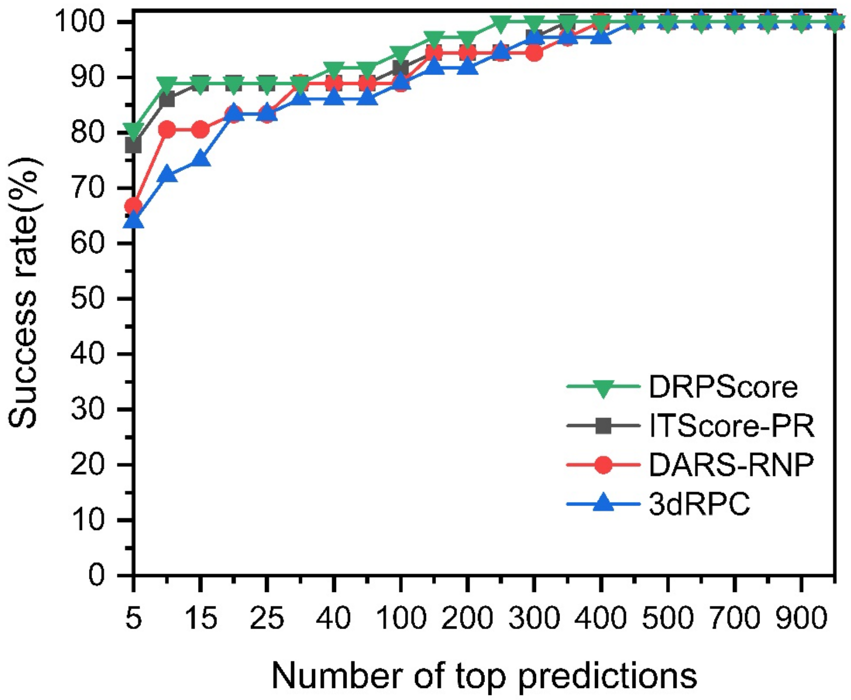

Supplementary Figure 4. Score-RMSD scatter plots. The examples of score-RMSD (Root Mean Square Deviation) scatter plots in testing set II. The Pearson correlation coefficients of score-RMSD are 0.60, 0.72, and 0.62, respectively. Source data are provided as a Source Data file.

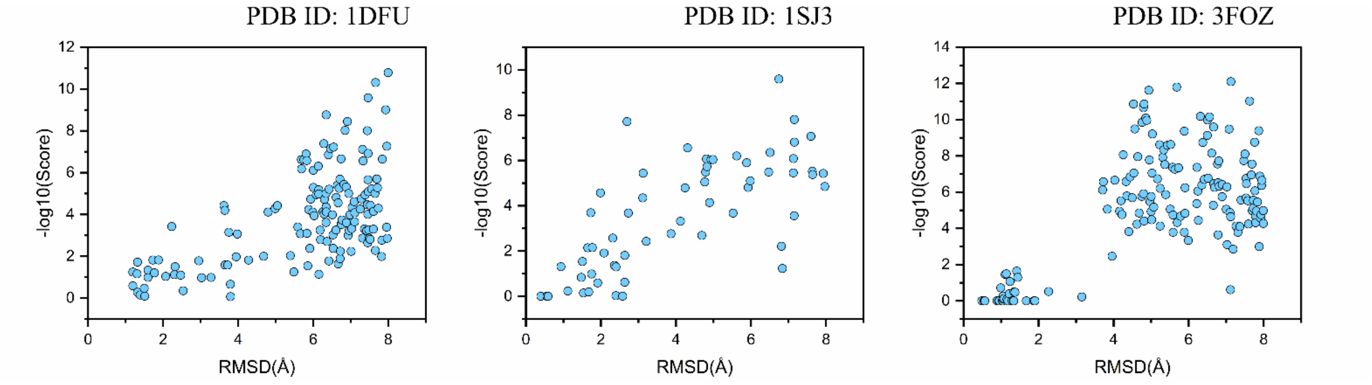

Supplementary Figure 5. The protein secondary structure. The protein secondary structure of RNA-protein complex predicted by PSIPRED (PDB ID:3MOJ).

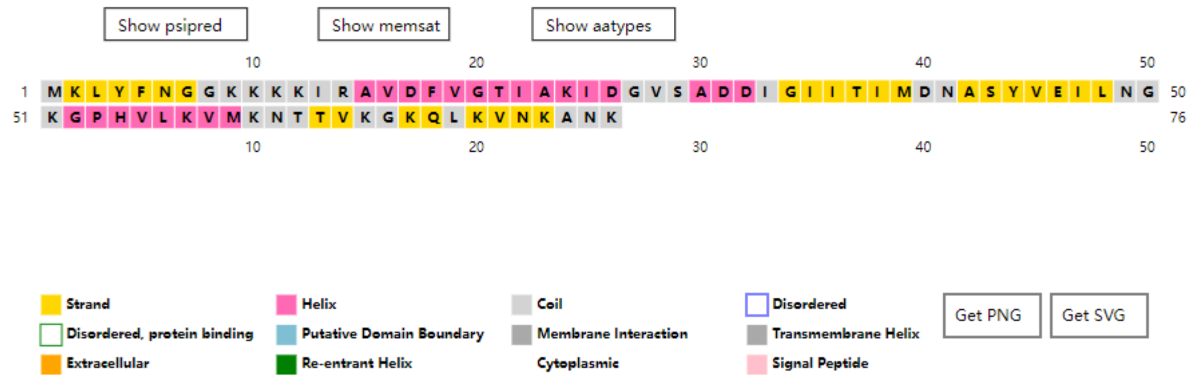

Supplementary Figure 6. The RNA secondary structure. The RNA secondary structure of RNA-protein complex predicted by forna (PDB ID:3MOJ).

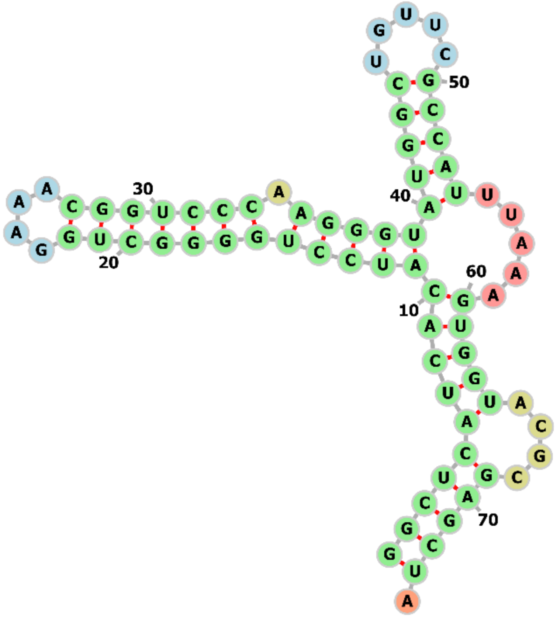

Supplementary Figure 7. The performance of DRPScore, 3DCNN and other scoring functions on the bound-bound testing sets. The average success rates and standard deviations (n = 3 independent testing) of DRPScore (green inverted triangle), 3DCNN (purple diamond), ITScore-PR (black square), DARS-RNP (red circle), and 3dRPC (blue triangle) on the bound-bound testing set. Data are presented as mean values +/- SD. Source data are provided as a Source Data file.

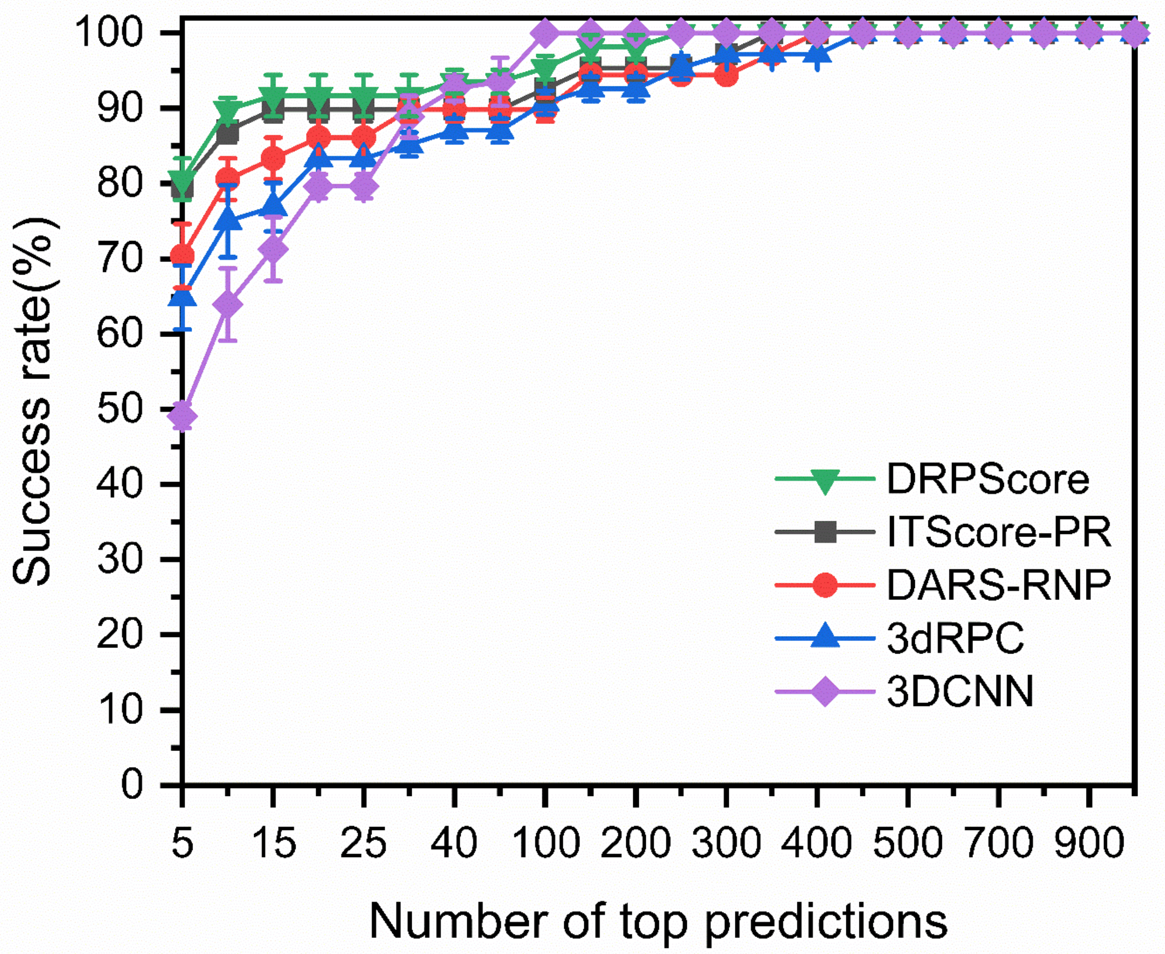

Supplementary Figure 8. The performance of DRPScore, 3DCNN and other scoring functions on the unbound-unbound testing sets. The success rates of DRPScore (colored in green), ITScore-PR (colored in orange), DARS-RNP (colored in purple), 3dRPC (colored in yellow) on the unbound-unbound testing set. Source data are provided as a Source Data file.

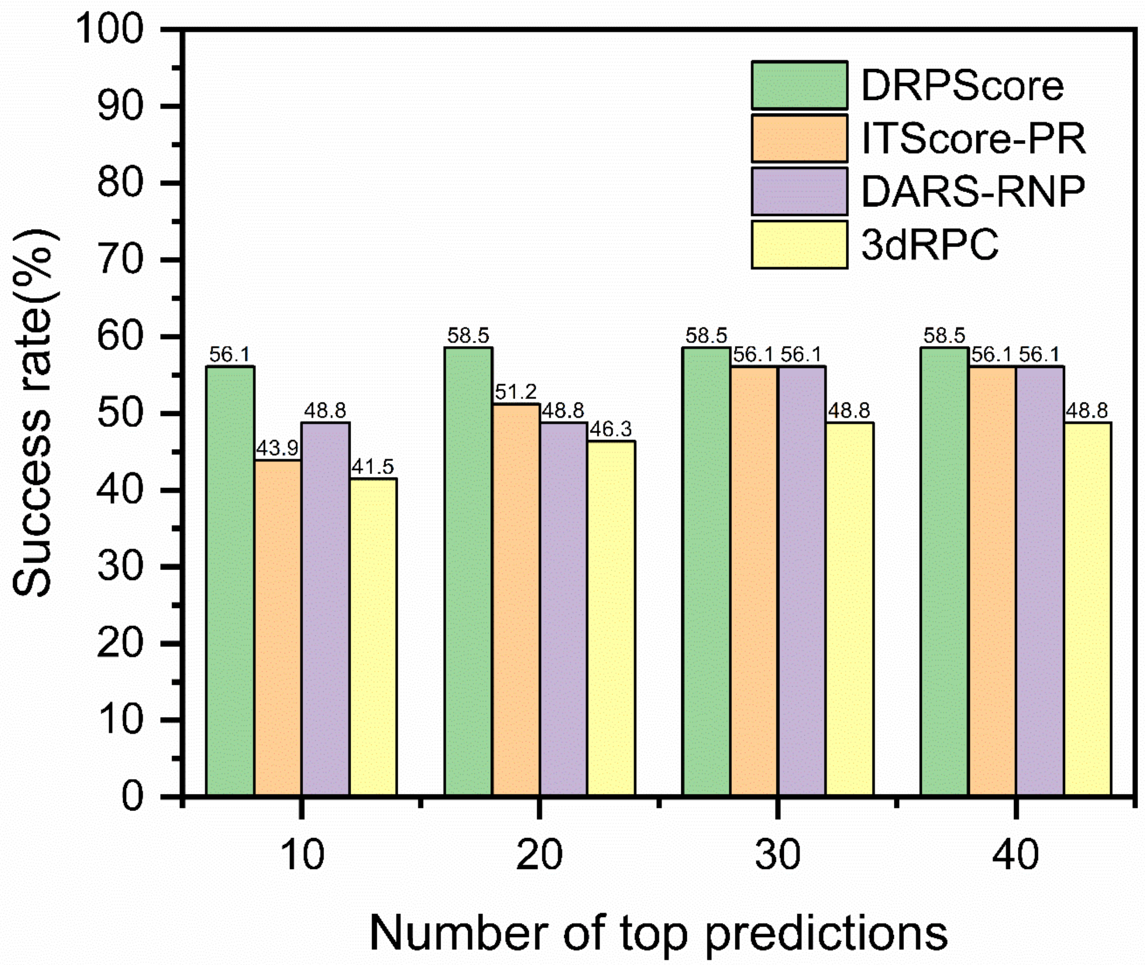

Supplementary Figure 9. The performance of DRPScore and other scoring functions on the entire unbound testing set II. The success rates of DRPScore (green inverted triangle), ITScore-PR (black square), DARS-RNP (red circle), and 3dRPC (blue triangle) on the entire unbound testing set II. Source data are provided as a Source Data file.

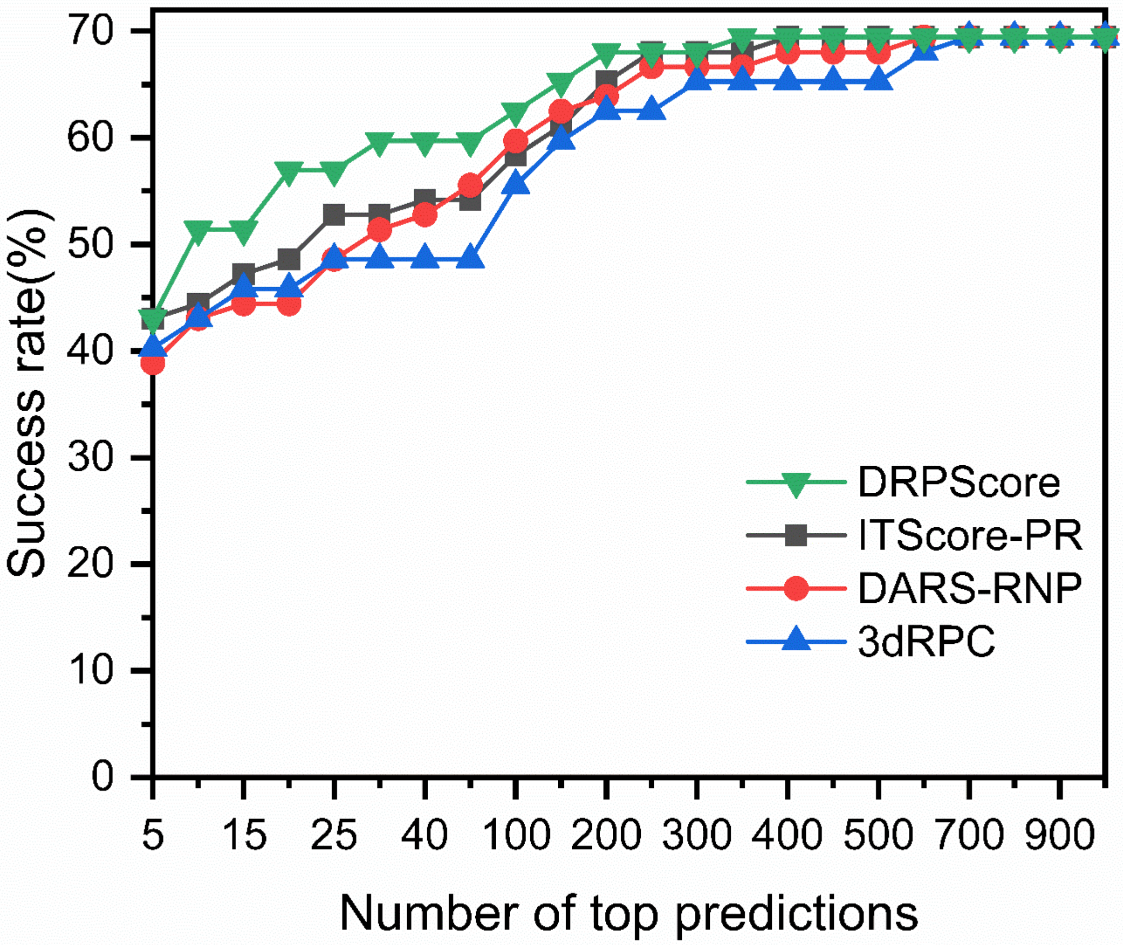

Supplementary Figure 10. Box plots of RMSD distribution. The distribution of RMSD of the top 10 structures selected by DRPScore (green circle) and the sum of three other scoring functions (ITScore-PR, DRPScore, 3dRPC) (Orange triangle) for the six examples on the testing set II with the sequence similarity cutoff of 0.8 (n = 10 structures for DRPScore, and n = 30 structures for the sum of other three scoring functions). The five lines from top to bottom of each box are: maximum value (exclude outliers), percentile line (75%), median value, percentile line (25%), and minimum value (exclude outliers). Source data are provided as a Source Data file.

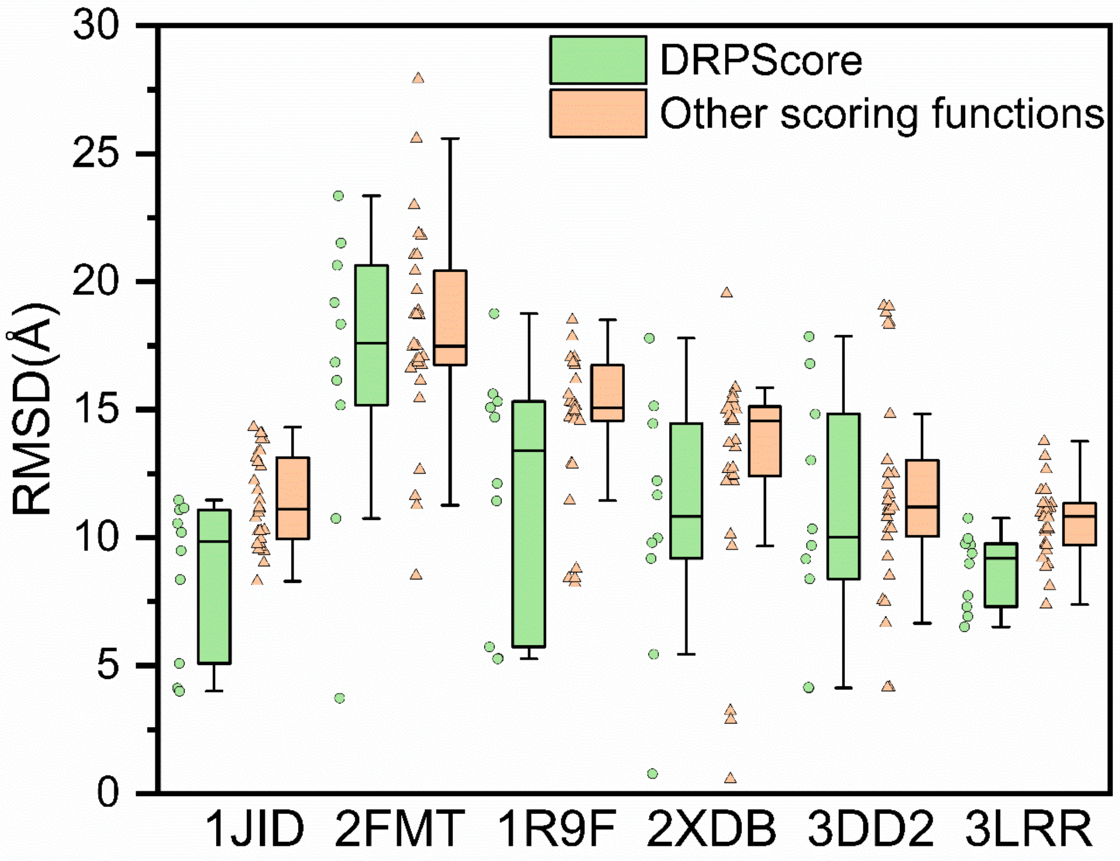

Supplement: Supplementary file 1 — Supplementary Information [file 41467_2023_36720_MOESM1_ESM.pdf]
