# Supplementary material for: Evaluating native-like structures of RNA-protein complexes through the deep learning method
Source: Nat Commun. 2023 Feb 24;14:1060. doi: 10.1038/s41467-023-36720-9 (PMC9958188; doi:10.1038/s41467-023-36720-9)
Supplement: Supplementary file 2 — Description of Additional Supplementary Files [file 41467_2023_36720_MOESM2_ESM.pdf]

<u>Description of Additional Supplementary Information Files Document</u>

File Name: Supplementary Data 1

Description: The ranking and interface RMSDs (Å) for the first successfully predicted RNA-protein complexes (RMSD < 4 Å) on the first independent bound-bound testing set.

File Name: Supplementary Data 2

Description: The ranking and interface RMSDs (Å) for the first successfully predicted RNA-protein complexes (RMSD < 4 Å) on the second independent bound-bound testing set.

File Name: Supplementary Data 3

Description: The ranking and interface RMSDs (Å) for the first successfully predicted RNA-protein complexes (RMSD < 4 Å) on the third independent bound-bound testing set.

File Name: Supplementary Data 4

Description: The ranking and interface RMSDs (Å) for the first successfully predicted RNA-protein complexes (RMSD < 4 Å) on the unbound testing set.

File Name: Supplementary Data 5

Description: The atom types and charges on RNA nucleotides.

File Name: Supplementary Data 6

Description: The atom types and charges on protein residues.

File Name: Supplementary Data 7

Description: The PDB IDs of the 277 RNA-protein complexes in the training set.

File Name: Supplementary Data 8

Description: The PDB IDs of three random independent bound-bound testing tests.

File Name: Supplementary Data 9

Description: The PDB IDs of the RNA-protein complexes for the unbound testing set.

File Name: Supplementary Data 10

Description: The minimum, maximum and average [illegible] of the first independent

bound-bound testing set generated by 3dRPC.

File Name: Supplementary Data 11

Description: The minimum, maximum and average [illegible] 0◌ள of the second independent bound-bound testing set generated by 3dRPC.

File Name: Supplementary Data 12

Description: The minimum, maximum and average [illegible] 0◌ள of the third independent bound-bound testing set generated by 3dRPC.

File Name: Supplementary Data 13

Description: The minimum, maximum, and average [illegible] 0◌ள of testing set II generated by 3dRPC.
