# Supplementary material for: Evaluating native-like structures of RNA-protein complexes through the deep learning method
Source: Nat Commun. 2023 Feb 24;14:1060. doi: 10.1038/s41467-023-36720-9 (PMC9958188; doi:10.1038/s41467-023-36720-9)
Supplement: Supplementary file 4 — Reporting Summary [file 41467_2023_36720_MOESM4_ESM.pdf]

nature portfolio

Corresponding author(s): YunJie Zhao
Last updated by author(s): Feb 5, 2023

Reporting Summary

Nature Portfolio wishes to improve the reproducibility of the work that we publish. This form provides structure for consistency and transparency in reporting. For further information on Nature Portfolio policies, see our Editorial Policies and the Editorial Policy Checklist.

Statistics

For all statistical analyses, confirm that the following items are present in the figure legend, table legend, main text, or Methods section.

n/a	Confirmed	
☐	☒	The exact sample size (*n*) for each experimental group/condition, given as a discrete number and unit of measurement
☒	☐	A statement on whether measurements were taken from distinct samples or whether the same sample was measured repeatedly
☒	☐	The statistical test(s) used AND whether they are one- or two-sided<br>*Only common tests should be described solely by name; describe more complex techniques in the Methods section.*
☒	☐	A description of all covariates tested
☒	☐	A description of any assumptions or corrections, such as tests of normality and adjustment for multiple comparisons
☐	☒	A full description of the statistical parameters including central tendency (e.g. means) or other basic estimates (e.g. regression coefficient) AND variation (e.g. standard deviation) or associated estimates of uncertainty (e.g. confidence intervals)
☒	☐	For null hypothesis testing, the test statistic (e.g. *F*, *t*, *r*) with confidence intervals, effect sizes, degrees of freedom and *P* value noted<br>*Give P values as exact values whenever suitable.*
☒	☐	For Bayesian analysis, information on the choice of priors and Markov chain Monte Carlo settings
☒	☐	For hierarchical and complex designs, identification of the appropriate level for tests and full reporting of outcomes
☐	☒	Estimates of effect sizes (e.g. Cohen's *d*, Pearson's *r*), indicating how they were calculated

Our web collection on statistics for biologists contains articles on many of the points above.

Software and code

Policy information about availability of computer code

Data collection	Nucleic Acid Database(NDB) (http://ndbserver.rutgers.edu/) for dataset downloading;<br>Zoulab (http://zoulab.dalton.missouri.edu/RNAbenchmark/)
Data analysis	3dRPC (http://biophy.hust.edu.cn/new/3dRPC) for data generation;<br>CD-HIT (web server https://www.bioinformatics.org/cd-hit/);<br>forna (web server http://rna.tbi.univie.ac.at/forna/);<br>PSIPRED (version 4.0 http://bioinf.cs.ucl.ac.uk/psipred/);<br>ITScore-PR (http://zoulab.dalton.missouri.edu/resources_itscorepr.html);<br>DARS-RNP (https://genesilico.pl/software);<br>HBPLUS (version 3.2 https://www.ebi.ac.uk/thornton-srv/software/HBPLUS/);<br>DRPScore (https://github.com/Zhaolab-GitHub/DRPScore/tree/master)

For manuscripts utilizing custom algorithms or software that are central to the research but not yet described in published literature, software must be made available to editors and reviewers. We strongly encourage code deposition in a community repository (e.g. GitHub). See the Nature Portfolio guidelines for submitting code & software for further information.

Data

Policy information about availability of data

All manuscripts must include a data availability statement. This statement should provide the following information, where applicable:

- Accession codes, unique identifiers, or web links for publicly available datasets
- A description of any restrictions on data availability
- For clinical datasets or third party data, please ensure that the statement adheres to our policy

A full list with links of the PDB codes used in this study is available in supplementary data 7-9. All data sets used in this paper can be downloaded from Nucleic Acid Database (http://ndbserver.rutgers.edu/) and Zoulab (http://zoulab.dalton.missouri.edu/RNAbenchmark/). The data that supports the findings of this study, including scoring function, training set, testing sets, and examples, are available to download at https://github.com/Zhaolab-GitHub/DRPScore_v1.0. The source data are provided with this paper.

Human research participants

Policy information about studies involving human research participants and Sex and Gender in Research.

Reporting on sex and gender	We didn't do the correlational research
Population characteristics	We didn't do the correlational research
Recruitment	We didn't do the correlational research
Ethics oversight	We didn't do the correlational research

Note that full information on the approval of the study protocol must also be provided in the manuscript.

Field-specific reporting

Please select the one below that is the best fit for your research. If you are not sure, read the appropriate sections before making your selection.

☒ Life sciences ☐ Behavioural & social sciences ☐ Ecological, evolutionary & environmental sciences

For a reference copy of the document with all sections, see nature.com/documents/nr-reporting-summary-flat.pdf

Life sciences study design

All studies must disclose on these points even when the disclosure is negative.

Sample size

We have done a large-scale analysis of the RNA-protein complex structures to generate a training set. The previous 3DCNN model for RNA structure prediction generated 300 decoys for one RNA structure. Here, we generate 500 decoys for each complex structure. To construct a diverse training dataset of RNA-protein complex structures, we extracted 951 available RNA-protein complex structures from the NDB database (before July 13, 2022) with the search options “only RNA and Protein” and “Resolution cutoff 3.5Å (X-ray)”. Second, we removed the short RNAs with lengths of less than 10 nucleotides. Third, we considered the cases with no more than six chains of protein or RNA as described in ITScore-PR. Fourth, we removed the RNA redundancy by 0.95 sequence similarity cutoff as RASP and DRNA using CD-HIT. Finally, we obtained a non-redundant RNA-protein dataset with 346 structures. We randomly selected 277 RNA-protein complex structures for training from the 346 non-redundant RNA-protein structures. The remaining RNA-protein complexes are further processed to build bound-bound testing sets. We used 3dRPC to generate RNA-protein structural decoys. 3dRPC first generates the RNA-protein complex by the RPDOCK algorithm and then evaluates the structures by RPRANK. For each RNA-protein complex in the training set, 10000 decoys were generated using the command of ‘3dRPC -mode 9 -system 8 -par RPDOCK.par’. Then, we calculated the RMSDs of the complex structures using the following command ‘3dRPC –mode 2 –system 0 –par RMSD.par’. Finally, we selected the top 500 structures from 10000 decoys by RMSD ranking. Thus, there are 1 native structure and 500 docking structures for each RNA-protein complex.

We generated 1000 decoys for each complex structure for the testing sets as in previous research (ITScore-PR and 3dRPC). Testing set I is the non-redundant bound-bound RNA-protein docking benchmark. We randomly selected 36 RNA-protein complexes from the remaining non-redundant RNA-protein complexes mentioned above. We generated three bound-bound RNA-protein sets for a fair comparison. Then, we generated 1000 decoys for each RNA-protein complex in those three sets by 3dRPC.

Testing set II is the non-redundant unbound RNA-protein docking benchmark provided by Huang and Zou. We removed the redundancy between training and testing sets II by 0.95 sequence similarity cutoff using CD-HIT. Thus, this benchmark remains 57 RNA-protein unbound complex structures, which consist of 41 unbound-unbound complexes and 16 unbound-bound complexes (Supplementary Table 11). For each RNA-protein complex in this benchmark, 1000 decoys were generated by 3dRPC using the command of ‘3dRPC -mode 9 -system 8 -par RPDOCK.par’. The relative RMSDs between decoys and native complex structures were calculated using the command of ‘3dRPC –mode 2 –system 0 –par RMSD.par’.

Data exclusions

We removed the redundancy between training and testing sets II by 0.95 sequence similarity cutoff using CD-HIT.

Replication	All results could be reproduced by the downloadable package of DRPScore based on the Methods/Supporting Information.
Randomization	The training set was collected from the NDB database by 0.95 sequence similarity cutoff using CD-HIT.<br>Testing set I of DRPScore was randomly collected from the remaining non-redundant structures for validation.<br>Testing set II is a recognized testing set provided by Huang and Zou. We used CD-HIT to remove RNA redundancy between training and testing set II by 0.95 sequence similarity cutoff.
Blinding	All the experiments are based on preexisting data from other sources (NDB database). The analysis is done by software without human intervention. Therefore, the results are not expected to be influenced by subjective factors.

Reporting for specific materials, systems and methods

We require information from authors about some types of materials, experimental systems and methods used in many studies. Here, indicate whether each material, system or method listed is relevant to your study. If you are not sure if a list item applies to your research, read the appropriate section before selecting a response.

Materials & experimental systems

n/a	Involved in the study
☒	☐ Antibodies
☒	☐ Eukaryotic cell lines
☒	☐ Palaeontology and archaeology
☒	☐ Animals and other organisms
☒	☐ Clinical data
☒	☐ Dual use research of concern

Methods

n/a	Involved in the study
☒	☐ ChIP-seq
☒	☐ Flow cytometry
☒	☐ MRI-based neuroimaging
